# Radiographic vs. MRI vs. arthroscopic assessment and grading of knee osteoarthritis - are we using appropriate imaging?

**DOI:** 10.1186/s40634-021-00442-y

**Published:** 2022-01-03

**Authors:** Samuel Newman, Huzefah Ahmed, Nader Rehmatullah

**Affiliations:** Wrightington, Wigan & Leigh NHS Foundation Trust, Wigan Lane, Wigan, WN1 2NN UK

**Keywords:** Radiographic, Radiograph, Kellgren-Lawrence, MRI, Arthroscopic, Arthroscopy, Outerbridge, Osteoarthritis, Knee

## Abstract

**Purpose:**

Radiographs and MRI scans are commonly used imaging techniques in the assessment of knee osteoarthritis. However, it currently remains uncertain how good a representation of the actual condition of the knee joint these investigations provide. By comparing them against arthroscopic findings the aim of our study was to conclude how accurate these imaging techniques are at grading knee osteoarthritis.

**Methods:**

This was a retrospective study looking at knee arthroscopies performed at a tertiary centre over a 5 year period. The Outerbridge grade given at arthroscopy was correlated with pre-operative radiograph and MRI scores, so as to assess the reliability of these imaging techniques at predicting the actual severity of knee osteoarthritis seen.

**Results:**

Kellgren-Lawrence (KL) grading of radiographs was moderately correlated with Outerbridge grades from arthroscopy for the medial compartment of the knee (Spearman’s rho (SR) 0.483, *p* < 0.001), with a milder correlation in the lateral compartment (SR 0.218, *p* = 0.003). MRI reporting of knee osteoarthritis was moderately correlated with Outerbridge grades in the medial compartment (SR 0.451, *p* < 0.001), mildly correlated for both the lateral (SR 0.299, *p* < 0.001) and patellofemoral joint compartments (SR 0.142, *p* = 0.054). KL and MRI grading was moderately correlated for the medial compartment (SR 0.475, *p* < 0.001) and mildly correlated for the lateral compartment (SR 0.277, *p* < 0.001).

**Conclusion:**

The ability of radiographs to represent the actual condition of knee osteoarthritis is underestimated. KL grading especially best represents the disease seen in the medial compartment of the knee joint, with a moderate correlation to Outerbridge scores given on arthroscopic assessment. We suggest that whilst MRI is a useful tool in the investigation of knee symptoms, it is often unnecessarily used in patients with OA, when in fact, radiographs alone would be sufficient.

**Evidence level:**

III

## Introduction

The knee is the most common joint affected by osteoarthritis (OA). Changes inside the joint such as cartilage loss, osteophyte formation, meniscal tears and loose bodies can lead to manifestation of the common symptoms of knee pain, swelling and reduced mobility. The pathophysiological processes that lead to these osteoarthritic changes in the knee are influenced by hereditary factors, ageing, excess loading of the joint, and mechanical injuries [1]. Trauma resulting in damage to the anterior cruciate ligament (ACL) or menisci also predisposes the knee to the development of OA. Ten to 20 years following an ACL rupture, 50% of patients will develop knee OA. A similar rate is seen in those followed up after surgical resection of meniscal tears [2].

Degenerative meniscal tears are a common manifestation of OA occurring in the knee. For these patients, who commonly present between 40 and 60 years of age, the arthritic process is likely to have started prior to meniscectomy. However, for traumatic tears which more commonly occur in the younger population, the knee more often than not has limited OA prior to injury and treatment. Therefore, as would be expected, the progression of osteoarthritis is greater in those who suffer degenerative meniscal tears compared to traumatic tears [2].

There is increasing evidence to suggest that patients with knee OA with degenerative meniscal tears who undergo an arthroscopic partial meniscectomy do not have significantly improved patient related outcome scores and more often than not require further invasive surgery soon after. As the meniscal tear is likely a result of OA in the knee in these cases, resection of the damaged meniscus does little to slow progression of, or relieve symptoms attributable to, the underlying disease process [3]. Comparative studies have shown that arthroscopic debridement and lavage, commonly performed in the same operation as meniscectomy, also demonstrated no superior improvement in patients’ reported pain when compared to placebo [4].

Other invasive management procedures for knee OA include realignment osteotomy and prosthetic joint replacement. The possible options for joint replacement include a unicompartmental knee replacement or a total knee replacement. When joint replacements fail then joint fusion may be used as a last resort to rescue some function of the limb [3, 4].

Imaging is one of the tools we use in assessing OA and associated connective tissue complications: helping us to identify when the surgical procedures aforementioned are indicated. Radiographs are the initial mode of imaging used to investigate an osteoarthritic knee. Changes indicating OA include joint space narrowing (JSN), osteophytes, subchondral sclerosis, cyst formation and bony deformity. Severity of OA can be assessed using these hallmark signs, with the classification system most widely used for such purposes being the Kellgren and Lawrence (KL) classification system (shown under methods in Table 1) [5].

**Table 1:** KL classification system for knee OA

Grade	Radiograph findings
0	No findings of OA
1	Doubtful narrowing of joint space & possible osteophytic lipping
2	Definite osteophytes & possible narrowing of joint space
3	Moderate multiple osteophytes, definite narrowing of joint space, small pseudocystic areas with sclerotic walls & possible deformity of bone contour
4	Large osteophytes, marked narrowing of joint space, severe sclerosis & definite deformity of bone contour

Magnetic resonance imaging (MRI) can be used to gain more information about the knee joint than is obtainable from radiographs alone. MRI can be used to eliminate other diagnoses, as well as give us a more detailed picture of the chondral surfaces and bone oedema caused by osteoarthritis. Clinically, this means that MRI scanning is used when pain or other symptoms are out of keeping with the severity of the radiograph findings and alternative diagnoses, such as synovitis, tendinitis and damage of cartilaginous structures, are suspected [6]. There is currently no gold standard classification system for assessing the MRI severity of knee osteoarthritis.

Knee pain in itself is a non-specific symptom that does not correlate well with radiographic evidence of OA. As such, radiographs should not be solely used to assess a patient’s OA or knee pain [7]. It is currently unclear if there is any correlation between the findings of knee OA on MRIs and radiographs. It does, however, appear that MRI may be more sensitive at picking up early OA changes, and that when followed up, progression of MRI changes are matched by radiographic changes [8, 9]. This does obviously depend on the MRI technique used. Assessment of cartilage condition can be done more accurately with newer techniques such as T2 weighting and delayed galodinium-enhanced MR imaging of cartilage [10, 11].

In clinical practice it is very useful to be able to visualise the condition of a joint with non-invasive imaging. Decisions can then be made confidently in regard to the appropriateness for surgical procedures when combined with the clinical history and examination findings. It is currently unclear if findings found on such imaging match those of direct inspection of a joints disease process (which can be classified at arthroscopy using the Outerbridge classification system seen under methods in Table 2) [12]. Studies have reported only a moderate correlation between the levels of osteoarthritis seen on radiograph with that seen at arthroscopy [13]. When comparing MRI findings to that of arthroscopy, some studies have concluded that MRI is unsuitable for assessing cartilage but has a function in identifying meniscus and ligament damage. The sensitivity of MRI varies depending on what anatomical structure is being assessed and the quality of the MRI scanner itself. Posterior horn tears of the medial meniscus were the most commonly correctly identified abnormality, with lower sensitivity for ACL and lateral meniscus tears [14].

**Table 2:** Modified Outerbridge classification

Modified Outerbridge score	Description of gross cartilage quality
0	Normal
1	Chondromalacia
2	Partial thickness fibrillation
3	Deep fibrillation
4	Full thickness cartilage loss

We have conducted this study to see if osteoarthritic changes of the knee seen on radiographs correlate with that of MRI scans, and furthermore, if the changes on these two commonly used types of imaging are representative of the actual condition of the joint seen at arthroscopy. In a time of increasing burden on healthcare services, it is important for clinicians to be efficient with resource allocation. By evidencing how accurately different imaging modalities are at representing the actual condition of the knee joint we can help to inform decisions as to whether further imaging is warranted, and in turn, prevent unnecessary use of radiological and surgical services.

## Materials and methods

A list was created of all arthroscopic knee surgeries, performed by orthopaedic surgeons at a tertiary centre, between January 2008 and June 2013. If more than one arthroscopy was performed on a patient’s knee in this time period then only the first arthroscopy was looked at, and any subsequent procedures were ignored. If a patient had another arthroscopy performed on the contralateral knee then the results from this procedure, as well as the ipsilateral knee procedure, were also included in the analysis. After these exclusions a total of 942 arthroscopic procedures remained eligible for the study.

A total of 793 of the 942 cases had pre-operative x-rays we were able to access. The radiographs in each case were reviewed by a junior trainee, under supervision from a senior orthopaedic registrar. Using the KL classification system, a grade was given for the severity of OA of both the medial and lateral aspects of the knee. This was done blinded to any knowledge of the results of MRI scans or intraoperative grading, so as to prevent influencing of the KL grade given. There was an inadequate number of participants with appropriate radiograph views to assess patellofemoral joint (PFJ) compartment disease.

A total of 452 of the 942 eligible cases had had pre-operative MRI scans of the knee. These had all previously been reported by a consultant radiologist. We collected information on the severity of OA seen in the medial, lateral and PFJ compartments on these scans from the radiologist’s report. OA changes present were reported as mild, moderate or severe. We therefore converted these to an ordinal grading system (see Table 3 below). It was also noted if a medial or lateral meniscus tear was reported, as well as any cruciate ligament injuries.

**Table 3:** Ordinal MRI grading for OA severity

Ordinal grading given	MRI reported OA severity
0	None
1	Mild
2	Moderate
3	Severe

We were able to access the online operation notes for 774 of the 942 eligible arthroscopies. From the operation notes we could record – or calculate if not written explicitly – the grade of OA seen in each compartment intraoperatively, using the Outerbridge classification system. It was also noted if a medial or lateral meniscus tear was seen, as well as any cruciate ligament injuries that were reported by the operating surgeon.

Using the statistical program JASP, analysis was performed on the cohort of patients who had pre-operatively undergone both forms of imaging mentioned above, as well as accessible operation notes. All the data sets were proven to be non-parametric using the shapro-wilks test. Therefore, Spearman’s rank correlation coefficient, or Spearman’s rho (SR), was calculated to assess the correlation between the extent of OA seen on pre-operative imaging techniques and that seen intraoperatively for each compartment for the 186 eligible patients.

## Results

The mean age of our study population was 56.9 years. Mechanical symptoms were the indication for arthroscopy for a total of 98 of the 186 patients. A total of 159 of the 186 of the patients were identified as having meniscal tears on MRI scanning. A total of 158 of these patients were in fact proved to have such pathology by arthroscopic assessment. One cruciate ligamentous injury was identified on MRI, whereas a total of 12 were identified at arthroscopy.

In Table 4 below the mean grade of arthritis identified on each imaging is reported. Table 5 contains the SR values representing correlation between the grades seen via different modalities for study participants.

**Table 4:** Mean grades of arthritis seen in knee compartment by imaging modality

	Medial compartment		Lateral Compartment		PFJ compartment	
	*Mean*	*SD*	*Mean*	*SD*	*Mean*	*SD*
**KL grade**	2.02	0.956	1.38	0.749		
**MRI grade**	0.77	0.993	0.62	0.885	0.49	0.815
**Outerbridge grade**	2.41	1.316	1.29	1.224	2.01	1.377

**Table 5:** Spearman’s Rho results with *p*-values

	Medial compartment		Lateral Compartment		PFJ compartment	
	*SR*	*p-value*	*SR*	*p-value*	*SR*	*p-value*
**X-ray vs MRI**	0.475	< 0.001	0.277	< 0.001		
**X-ray vs arthroscopy**	0.483	< 0.001	0.218	0.003		
**MRI vs arthroscopy**	0.451	< 0.001	0.299	< 0.001	0.143	0.054

## Discussion

Smaller-numbered studies have previously shown a relationship between OA changes seen on radiographs and MRI of the knee. Mild to moderate correlations were reported between KL grades and cartilage composition seen throughout the knee on MRI scans in 93 postmenopausal women, and a strong correlation was reported between KL grades and whole-organ magnetic resonance imaging scores (WORMS) in 72 subjects selected from the multicenter osteoarthritis study [15, 16]. In a larger study of 724 knees, strong correlations between KL grade and MRI identified osteophytes were seen throughout the three compartments. Bone marrow lesions and meniscal extrusions were also highly correlated with KL grade in the medial compartment, with less of a relation being seen for these findings in the lateral and PFJ compartments [17]. This report of a stronger association between radiograph and MRI findings in the medial compartment is contradictory to other studies, which have in fact found that there is a greater correlation between findings in the lateral compartment on these two forms of imaging [15, 18].

In our study’s patient population, the OA grades given for knee radiographs were moderately matched to those reported in the medial compartment on MRI scan, with a milder correlation seen with those in the lateral compartment [19]. The KL grades though, were in fact, mildly better predictors for the severity of OA seen in the medial compartment at arthroscopy than those reported on MRI scans. The reverse was true for the lateral compartment, with MRI reports giving a slightly better indicator of the Outerbridge grade seen, compared to radiographic assessment. MRI correlation with Outerbridge grades for the PFJ was weaker than that seen in the other 2 compartments.

Previous smaller studies conducted with 117 subjects in Asia, and 125 subjects in America, reported spearman’s correlation coefficients between KL and Outerbridge grades for the whole knee of 0.32 and 0.49 respectively [20, 21]. A larger study conducted by the MARS group found an even smaller correlation coefficient of 0.3 between Outerbridge and KL grade on anteroposterior view radiographs in 594 patients. However, it did report a correlation coefficient of 0.42 for Rosenberg view radiographs in the 416 subjects that had undergone these [13]. In fact, weight-bearing radiographs taken from a posteroanterior view with the knee in 45 degrees of flexion (known as the Rosenberg view) have been shown in several studies to have a higher sensitivity for detecting OA than standing AP views [22]. Our study assessed the correlation for each individual compartment of the knee. We found that KL grading better represents the actual OA changes seen in the medial compartment, than has previously been reported by other studies which have combined all three compartments. In turn, our results suggest that KL grading is less reliable for the lateral compartment of the knee than these other studies.

### Limitations of study

We acknowledge that radiographic assessment has its limitations, and that whichever grading system is used, simple imaging can miss subtle changes in the knee that occur in the early stages of OA [23–25]. Some criticisms of the KL classification system specifically are that it can overemphasise the importance of osteophytes, and ignores the true importance of joint space narrowing, in its grading of OA [24, 26]. It is important to identify early OA changes, as long-term follow-up has shown that those identified on radiographs as KL grade 1 were much more likely to have a progression of OA than those identified as KL grade 0 [26]. In addition to being useful for identification of soft tissue knee injuries, another purpose of investigation with MRI scanning is that it can be used to identify these early OA changes that are missed by radiographs [9].

In addition to the limitations of the KL grading system mentioned above, we are also aware that our results could have been affected by the subjectivity of its interpretation. We acknowledge that although assessment of knee radiographs is fairly simple, in our study this was done by a junior trainee and not an expert (although they were trained in the first instance by a senior and experienced colleague). And, although the MRI reports used by our study were reported by experts, the findings in them are still open to subjectivity between radiologists. This is in part due to human factors, as well as the lack of a gold standard classification system for knee OA on MRI scans. In addition there may be indiscrepancies in the reporting caused by different specialty clinicians reporting the different imaging types (an orthopaedic trainee interpreting x-rays and radiologists reporting MRIs). Although interpretation of the radiographs was performed blind (with no knowledge of other imaging or arthroscopy results) interpretation of MRI scans by the radiologists was likely done after viewing all relevant imaging.

The MRI scans used in our study were performed with standard sequences only, and therefore may not be the best type for analysis of cartilage changes occurring in knee OA. However, since more complex sequence MRIs are not necessarily in widespread use throughout clinical settings, our findings regarding the reliability of standard MRI in assessing the condition of an osteoarthritic knee is still useful information for clinicians who wish to do so.

## Conclusion

It is our view that radiographs are an underrated imaging technique for arthritis of the knee. They are surprisingly underused in the investigation of knee symptoms, despite having been shown to be reliable enough to diagnose OA without the need for further imaging such as MRI scans [27]. Furthermore, when using a combination of radiograph views, clinicians are even able to accurately predict the suitability of a patient for knee arthroplasty surgery [28]. It is becoming more important to have a good representation of the condition of osteoarthritis in the knee joint via imaging, as arthroscopy becomes less commonplace. This fall in favour for arthroscopies follows several systematic reviews which concluded that knee arthroscopy provides little benefit in terms of long-term pain and function [29–31]. As such, it is going to become more customary that the knee joint has not been actually visualized by a surgeon prior to procedures such as arthroplasty, and therefore radiographs will be required to provide the best likeness of what to expect intraoperatively.

## Data Availability

The datasets used and/or analysed during the current study are available from the corresponding author on reasonable request.

## Abbreviations

OA: Osteoarthritis
ACL: Anterior cruciate ligament
JSN: Joint space narrowing
KL: Kellgren-Lawrence
MRI: Magnetic resonance imaging
PFJ: Patellofemoral joint
SR: Spearman’s rho
